# Activation of liver X receptor attenuates lysophosphatidylcholine‐induced IL‐8 expression in endothelial cells *via* the NF‐κB pathway and SUMOylation

**DOI:** 10.1111/jcmm.12903

**Published:** 2016-08-04

**Authors:** Xukun Bi, Jiale Song, Jing Gao, Juanjuan Zhao, Meihui Wang, Corey A. Scipione, Marlys L. Koschinsky, Zhao V. Wang, Shiming Xu, Guosheng Fu

**Affiliations:** ^1^ Department of CardiologyBiomedical Research (Therapy) CenterSir Run Run Shaw HospitalSchool of MedicineZhejiang UniversityHangzhouZhejiang ProvinceChina; ^2^ Institute of Translational MedicineSchool of MedicineZhejiang UniversityHangzhouZhejiang ProvinceChina; ^3^ Department of Chemistry and BiochemistryUniversity of WindsorWindsorONCanada; ^4^ Division of CardiologyDepartment of Internal MedicineUniversity of Texas Southwestern Medical CenterDallasTXUSA; ^5^ Present address: Robarts Research InstituteWestern UniversityLondonONCanada

**Keywords:** liver X receptor, lysophosphatidylcholine, NF‐κB, SUMOylation

## Abstract

The liver X receptor (LXR) is a cholesterol‐sensing nuclear receptor that has an established function in lipid metabolism; however, its role in inflammation is elusive. In this study, we showed that the LXR agonist GW3965 exhibited potent anti‐inflammatory activity by suppressing the firm adhesion of monocytes to endothelial cells. To further address the mechanisms underlying the inhibition of inflammatory cell infiltration, we evaluated the effects of LXR agonist on interleukin‐8 (IL‐8) secretion and nuclear factor‐kappa B (NF‐κB) activation in human umbilical vein endothelial cells (HUVECs). The LXR agonist significantly inhibited lysophosphatidylcholine (LPC)‐induced IL‐8 production in a dose‐dependent manner without appreciable cytotoxicity. Western blotting and the NF‐κB transcription activity assay showed that the LXR agonist inhibited p65 binding to the IL‐8 promoter in LPC‐stimulated HUVECs. Interestingly, knockdown of the indispensable small ubiquitin‐like modifier (SUMO) ligases Ubc9 and Histone deacetylase 4 (HDAC4) reversed the increase in IL‐8 induced by LPC. Furthermore, the LPC‐induced degradation of inhibitory κBα was delayed under the conditions of deficient SUMOylation or the treatment of LXR agonist. After enhancing SUMOylation by knockdown SUMO‐specific protease Sentrin‐specific protease 1 (SENP1), the inhibition of GW3965 was rescued on LPC‐mediated IL‐8 expression. These findings indicate that LXR‐mediated inflammatory gene repression correlates to the suppression of NF‐κB pathway and SUMOylation. Our results suggest that LXR agonist exerts the anti‐atherosclerotic role by attenuation of the NF‐κB pathway in endothelial cells.

## Introduction

Atherosclerosis is the principle pathological feature underlying coronary artery disease 1. It is an inflammatory process initiated by endothelial dysfunction and structural alterations that permit low‐density lipoproteins (LDLs) to accumulate in the sub‐endothelium, where they are susceptible to oxidative modification. Oxidized LDL (ox‐LDL) triggers endothelial cells to secret adhesion molecules and chemokines, thereby driving monocytes to form early fatty‐streak lesions during the initial stage of atherogenesis. As a major chemotactic lipid component of ox‐LDL, lysophosphatidylcholine (LPC) plays an important role in the recruitment of monocytes/macrophages 2; this process involves a sequence of rolling, firm adhesion, migration and transendothelial diapedesis that is controlled by chemokines 3. Interleukin‐8 (IL‐8) is a prominent chemokine that triggers the firm adhesion of monocytes to the vascular endothelium 4, 5. And the influx of inflammatory cells through endothelium into a plaque is a critical process for atheroprogression 6, which is the target of multiple therapeutic approaches in current clinical applications.

Liver X receptor (LXRα and LXRβ) is a ligand‐dependent nuclear receptor that controls diverse pathways involved in development, reproduction, metabolism and inflammation 7. Liver X receptor α is primarily expressed in highly active metabolic cells, including hepatocytes, intestinal enterocytes, macrophages and adipocytes, whereas LXRβ is ubiquitously expressed 8. Liver X receptor promotes reverse cholesterol transport by directly increasing the transcription of genes involved in cholesterol efflux pathway, such as the ATP‐binding cassette A1 9, 10, ATP‐binding cassette transporter superfamily G member 1 (ABCG1) 11 and apolipoprotein E (apoE) 12. These cholesterol delivery processes from peripheral tissues to liver for excretion may reduce the atherosclerotic risk. Consistently, studies performed with atherosclerotic mouse models showed that the LXR agonist GW3965 strongly reduced lesion formation 13, 14, 15.

However, the effect of LXR pathway is less well‐established in endothelial cells, an important cell type that may contribute to the anti‐atherosclerotic effect induced by LXR agonist. Both LXRα and LXRβ are expressed in endothelial cells 16, 17. In atheroprotective thoracic aorta, expression of LXRα, LXRβ and their target genes are 5‐fold higher than atheroprone regions including aorta arch 18. Liver X receptor β represents the most important LXR isoform in endothelial cells. Activating of LXRβ prevents endothelial cellular senescence by up‐regulating the target gene sterol regulatory element binding protein‐1 19. Besides, LXRβ activation reduces overproduction of reactive oxygen species and increases endothelial nitric oxide synthase activity, which stimulates the migration of endothelial cells and preserves endothelial integrity 20. These studies suggest that LXR may play an important role in preventing endothelial dysfunction in the progression of atherosclerosis.

Liver X receptors exert the anti‐inflammatory effect by suppression of the Toll‐Like Receptor pathway 21. Liver X receptor agonist T0901317 and GW3965 can attenuate lipopolysaccharide‐induced expression of vascular cell adhesion endothelial‐1 and intracellular adhesion molecule‐1 16. The mechanism underlying the suppression of inflammatory genes by LXR is poorly understood. The LXR agonist can inhibit the nuclear entry of nuclear factor‐kappa B (NF‐κB) by inhibiting the phosphorylation and ubiquitin‐dependent degradation of the inhibitory κB (IκB) proteins 9. Interestingly, without altering the binding of NF‐κB to the DNA element or attenuating IκB degradation, LXR agonist can repression NF‐κB activation 22. These studies suggest the mechanism underlying LXRs' suppressive effects may involve more complex modifications.

In this study, we set up to investigate the anti‐inflammatory effect of LXR in endothelial cells. We showed that the LXR agonist inhibited the adhesion of monocytes to endothelial cells. The LXR agonist suppressed the expression of IL‐8 by LPC‐stimulated human umbilical vein endothelial cells (HUVECs) *via* the NF‐κB pathway and SUMOylation.

## Materials and methods

### Materials

L‐α‐LPC and GW3965 were purchased from Sigma‐Aldrich (St. Louis, Mo, USA). Lysophosphatidylcholine was dissolved in PBS and stored at −20°C, and the synthetic LXR ligand GW3965 was dissolved in Dimethyl Sulfoxide (DMSO).

### Cell culture

Human umbilical vein endothelial cells were obtained from ScienCell (Carlsbad, CA, USA) and cultured in endothelial cell medium (ECM) supplemented with 10% foetal bovine serum (FBS). For experimental treatments, cells (passages 3–8) were grown to 70–90% confluence. Then, they were incubated for 6 hrs with ECM containing 0.5% FBS, followed by the same medium supplemented with GW3965, LPC or vehicle. Equal volume of DMSO was used as the vehicle control for GW3965 and equal volume of PBS was used as the vehicle control for LPC. THP‐1 cells were human monocyte cells derived from the peripheral blood of the 1‐year‐old male infant with acute monocytic leukaemia disease. The cells were obtained from ATCC (Manassas, VA, USA) and were a kind gift from the state key laboratory for infectious diseases of Zhejiang University. Hela cells (ATCC) were grown in DMEM (Sigma‐Aldrich) supplemented with 10% FBS and antibiotics.

### Cell viability assay

A cell counting kit‐8 (CCK‐8; Dojindo, Kumamoto, Japan) was used as an indicator of cell viability. Briefly, water‐soluble tetrazolium salt is reduced by dehydrogenase activity in the cells to result in a yellow coloured formazan dye. Cells were seeded (0.5 × 10^4^ cells per well) into a 96‐well plate. After overnight incubation, cells were treated with different concentrations of LPC (0, 40, 60 or 80 μM) for 3 hrs to assess LPC cytotoxicity. To evaluate the role of LXR agonist, cells were pretreated with GW3965 (0.05, 2.5 or 5 μM) for 24 hrs and then incubated with 40 μM of LPC for 3 hrs. Six duplicate wells were examined in each group. For quantitative analysis of cell viability, 10 μl of the CCK‐8 solution was added to each well. After incubation at 37°C for 4 hrs in a humidified CO_2_ incubator, absorbance at 450 nm was monitored with a microplate reader (BioTek, Winooski, VT, USA). The values obtained were normalized to control cells incubated with vehicle alone. The experiments were performed four times.

### ELISA

Human umbilical vein endothelial cells were cultured in six‐well plates. Cell culture supernatants from all treatment conditions were collected and centrifuged (250 g for 10 min. at 4°C) to remove cell debris. The IL‐8 protein concentration was measured immediately using an ELISA Kit (R&D, Minneapolis, MN, USA) according to the manufacturer's recommendations. Protein content was used to normalize all treatments. The experiments were performed three times.

### NF‐κB transcription factor activity assay

Human umbilical vein endothelial cells were plated in 100‐mm dishes and treated with vehicle or 2.5 μM GW3965 for 24 hrs and then stimulated with PBS or 40 μM of LPC for 20 min. After incubation, nuclear proteins were isolated using the Nuclear Extraction Kit (Cayman Chemical, Ann Arbor, MI, USA). Specific binding of NF‐κB in the nuclear extract to the NF‐κB response element was detected with NF‐κB (p65) Transcription Factor Assay Kit (Cayman Chemical). Briefly, 10 μl of nuclear extract was added to wells coated with a specific double‐stranded DNA sequence; then, 90 μl of complete transcription factor buffer was added. Blank wells, a positive control, non‐specific binding wells and competitor dsDNA wells were all included on the plate. After the overnight incubation at 4°C, the wells were washed five times and incubated with NF‐κB (p65) antibody (except for the blank well) for 1 hr at room temperature. Then, the wells were washed five times and incubated with an HRP‐conjugated secondary antibody (except for the blank wells) for 1 hr at room temperature. Each well was washed again, and 100 μl of developing solution was added. After 45 min. incubation with gentle agitation, stop solution (100 μl) was added. The absorbance was read at 450 nm. The experiments were performed three times.

### Dual‐luciferase reporter assay

Hela cells were cultured in six‐well plates. After overnight incubation, cells were cotransfected with 0.5 μg of the NF‐κB luciferase reporter pNF‐κB‐TA‐luc, empty pGL3‐Basic plasmid, the −162 IL‐8 reporter construct or mutant construct, together with 0.5 μg of the Renilla luciferase reporter pRL‐TK (Promega, Madison, WI, USA) using the Lipofectamine 2000 DNA Transfection Reagent (Invitrogen, Carlsbad, CA, USA). The pNF‐κB‐TA‐luc was obtained from Beyotime Biotechnology (Shanghai, China). The empty pGL3‐Basic plasmid, the −162 IL‐8 reporter construct, −162 IL‐8 ΔAP‐1 and −162 IL‐8 ΔNF‐κB mutant constructs were used. After appropriate treatments, both the firefly and Renilla luciferase activities were measured using the Dual‐Luciferase Reporter Assay (Promega). Relative light units were calculated by normalizing to the Renilla luciferase activity. The experiments were performed three times.

### Immunofluorescence staining

To test the localization of NF‐κB, HUVECs were pretreated with 2.5 μM of GW3965 for 24 hrs and then stimulated with LPC (40 μM) for 0.5 hr. Cells on glass coverslips were fixed with 4% paraformaldehyde and permeabilized with 0.2% Triton X‐100 for 5 min. Coverslips were then incubated with the anti‐p65 antibody (1:100 dilution; Cell Signaling Technology, Beverly, MA, USA) overnight at 4°C. After washing, cells were incubated with Cy3 conjugated goat anti‐rabbit IgG (1:2000 dilution; Abcam, Cambridge, MA, USA). Coverslips were then mounted in glycerol and photographed with a fluorescence photomicroscope (Leica, Wetzlar, Germany).

### Western blotting analysis

Human umbilical vein endothelial cells were seeded in six‐well plates. After reaching confluence, HUVECs were treated with vehicle or 2.5 μM of GW3965 for 24 hrs and then stimulated with PBS or 40 μM of LPC for 5 min. to assess the phosphorylation of IκB kinase (IKK) and IκBα. To examine the degradation of IκBα, cells were stimulated by LPC (40 μM) for 0, 15, 30, 60 min. after transfecting with siUbc9 or treating with LXR agonist. Cells were then scraped into RIPA lysis buffer (Beyotime, Shanghai, China) supplemented with 1 mM phenylmethylsulfonyl fluoride. Equal amounts of proteins were separated on 10% sodium dodecyl sulphate polyacrylamide gels and electrotransferred to polyvinylidene difluoride membranes (Bio‐Rad, Hercules, CA, USA). The membranes were blocked with 5% non‐fat milk in Tris buffer solution for 1 hr at room temperature and incubated overnight with primary antibodies. The rabbit monoclonal anti‐phospho‐IκBα antibody (Ser32), mouse monoclonal anti‐IκBα antibody and anti‐GAPDH antibody (1:1000 dilution) and the secondary anti‐rabbit antibody (1:5000 dilution) were purchased from Cell Signaling Technology. The rabbit polyclonal anti‐phospho‐IKKα (S176) antibody, rabbit monoclonal anti‐IKKα, rabbit polyclonal anti‐phospho‐IKKβ (Y199) and rabbit monoclonal anti‐IKKβ were purchased from Abcam. Membranes were washed extensively in Tris‐buffered saline containing 0.1% (v/v) Tween‐20 prior to incubation for 1 hr with a secondary antibody conjugated to horseradish peroxidase. The bands were visualized with an enhanced chemiluminescence solution (Amersham, Haemek, Israel) on an LAS‐4000 image reader (Fujifilm, Tokyo, Japan), and band intensity was quantified by the Multi‐Gauge Software (Fujifilm, Tokyo, Japan). To ensure equal loading, protein levels were normalized to the levels of GAPDH.

### Transient transfection

Human umbilical vein endothelial cells were seeded into six‐well plates containing 2 ml of ECM for 24 hrs. Cells were then transfected with a negative control or siRNA (GenePharma, Shanghai, China) directed against Ubc9, HDAC4, SENP1, LXRα or LXRβ using the Hiperfect transfection reagent (Qiagen, Hilden, Germany). Briefly, siRNA was diluted to 100 pmol in 12 μl of Hiperfect and 500 μl of ECM without serum. The complexes were added to cells after 5–10 min. of incubation at room temperature. An additional 1.5 ml ECM supplemented with 10% FBS was added after 3 hrs. Cells were used for experiments after 24 hrs of incubation, and gene knockdown was validated by quantitative real‐time PCR.

### Quantitative real‐time PCR

Total RNA was extracted using TRIzol (Invitrogen) and then reverse transcribed to cDNA using the PrimeScript^™^ RT Master Mix (TaKaRa, DaLian, China). Real‐time PCR was performed on the Applied Biosystems 7500 real‐time PCR system using the SsoFast^™^ EvaGreen Supermix with Low ROX (Bio‐Rad). Values were normalized with GAPDH content. The primers for *IL‐8*,* LXR*α*, LXR*β*, Ubc9*,* HDAC4* and *SENP1* were as the following: *IL‐8* sense primer 5′‐CCTGATTTCTGCAGCTCTGT‐3′, antisense primer 5′‐AACTTCTCCACAACCCTCTG‐3′; *LXR*α sense primer 5′‐CTGTGCCTGACATTCCTCCT‐3′, antisense primer 5′‐GCATCCTGGCTTCCTCTCT‐3′; *LXR*β sense primer 5′‐CCTCTCCTACCACGAGTTCC‐3′, antisense primer 5′‐CGGACCCTCCTCCTTTACA‐3′; *Ubc9* sense primer 5′‐CAGGTCAGCATCTCGGTTC‐3′, antisense primer 5′‐CGCCTCCTCTCACTTTCAAT‐3′; *HDAC4* sense primer 5′‐AGAATGGCTTTGCTGTGGTC‐3′, antisense primer 5′‐ATCTTGCTCACGCTCAACCT‐3′; *SENP1* sense primer 5′‐AAATGCTCCCTTCGCTTCTC‐3′, antisense primer 5′‐AGTCGCTAGGTGGCTGAAGA‐3′; *GAPDH* sense primer 5′‐GGGTGTGAACCATGAGAAGT‐3′ and antisense primer 5′‐GACTGTGGTCATGAGTCCT‐3′. The fold change of relative mRNA expression was calculated using the 2^−∆∆Ct^ method.

### Adhesion assay

Adhesion of THP‐1 cells to the HUVEC monolayer was assessed as previously described 23. Briefly, HUVECs were seeded in six‐well plates and cultured in ECM containing 10% FBS. Cells were incubated with 2.5 μM of GW3965 for 24 hrs and then treated with 40 μM of LPC for 3 hrs. THP‐1 cells were washed three times with serum‐free ECM and suspended in serum‐free ECM. Approximately 1 ml of cells (20,000 cells/ml) was added to a well. In neutralization experiments, 0.4 μg/ml CXCL8/IL8 antibodies was added into the well. The human CXCL8/IL8 antibodies were purchased from R&D. After 20 min. of incubation, unfixed cells in wells were removed by three washes with serum‐free ECM. The adherent cells were counted in five randomly selected optical fields in each well.

### Statistical analysis

All experiments were performed at least three times. Data are expressed as the means ± S.E.M. One‐way or two‐way anova was used to compare the differences among multiple groups. Bonferroni corrections were used to indicate the differences between the mean values of paired groups among multiple groups. *P* < 0.05 was considered as statistically significant.

## Results

### LPC‐induced IL‐8 expression in HUVECs

**Figure 1:**
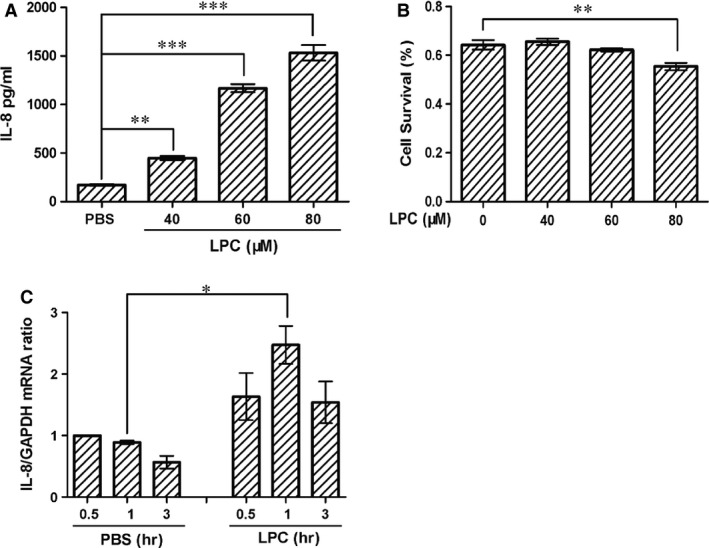
LPC‐induced IL‐8 expression. (**A**) HUVECs were treated with LPC (0, 40, 60 or 80 μM) for 3 hrs. IL‐8 was analysed by ELISA. (**B**) HUVECs were treated with the indicated concentrations of LPC for 3 hrs, and cell viability was determined. Cytotoxic assessment of HUVECs was measured by the CCK‐8 assay. (**C**) HUVECs were treated with 40 μM LPC for 0.5, 1 and 3 hrs. IL‐8 mRNA was analysed by real‐time PCR. *P < 0.05; **P < 0.01; ***P < 0.001. One‐way anova was used to compare the differences in **A** and **B**. Two‐way anova was used to compare the differences in **C**.

To assess the effect of LPC on IL‐8 production, HUVECs were stimulated with LPC at concentrations of 40, 60 and 80 μM. After 3 hrs of treatment, production of IL‐8 increased in a dose‐dependent manner (Fig. 1A). In response to 40 μM of LPC, IL‐8 levels significantly increased 2.49 ± 0.18‐fold compared with the control (*P* < 0.01). To evaluate any confounding issue of cell toxicity, cell survival at different concentrations of LPC was evaluated using CCK‐8 assay. Lysophosphatidylcholine‐induced cell toxicity was negligible at concentrations of 40–60 μM for 3 hrs (Fig. 1B). In the following experiments, we chose 40 μM of LPC for treatment in HUVECs. At the mRNA level, we found that IL‐8 mRNA expression reached a maximum at 1 hr of LPC treatment (Fig. 1C).

### LXR agonist inhibited LPC‐induced IL‐8 production in HUVECs

**Figure 2:**
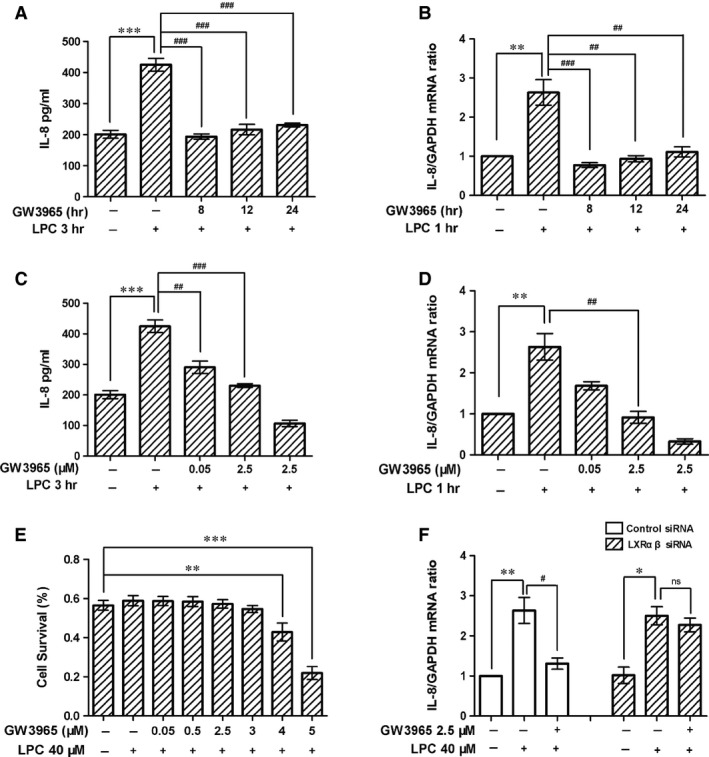
LXR agonist inhibited LPC‐induced IL‐8 production in HUVECs. (**A** and **B**) HUVECs were pretreated with 2.5 μM of GW3965 for 8, 12 or 24 hrs and then treated with 40 μM of LPC. IL‐8 protein and mRNA levels were analysed. (**C** and **D**) HUVECs were pretreated with GW3965 (0.05 or 2.5 μM) for 24 hrs and then treated with 40 μM of LPC. The level of the IL‐8 protein in the supernatant was measured by ELISA, and IL‐8 mRNA was analysed by real‐time PCR. (**E**) HUVECs were pretreated with GW3965 (0.05, 0.5, 2.5, 3, 4 or 5 μM) for 24 hrs and then treated with 40 μM of LPC for 3 hrs and cell viability was determined. (**F**) LXRαβ knockdown cells were under the stimulation of LPC or vehicle for 1 hr after incubation with or without GW3965, and IL‐8 gene expression was determined by real‐time PCR. *P < 0.05; **P < 0.01; ***P < 0.001, compared with the control. ^#^ *P* < 0.05; ^##^ *P* < 0.01; ^###^ *P* < 0.001, compared with the LPC‐treated group. One‐way anova was used to compare the differences.

To evaluate the effect of the LXR agonist on LPC‐induced IL‐8 production, we chose GW3965, which is a LXR agonist targeting both LXRα and LXRβ. Human umbilical vein endothelial cells were first treated with GW3965 for different time (8, 12 and 24 hrs). In the last 3 hrs of incubation, cells were treated with LPC (40 μM). We found that administration of GW3965 for as short as 8 hrs led to dramatic reduction in LPC‐mediated increase in IL‐8 and this effect persisted at the time‐point of 12 and 24 hrs (Fig. 2A). The changes in IL‐8 protein levels were consistent with reduction in mRNA levels after GW3965 treatment (Fig. 2B). After establishing the time‐dependent response of GW3965, we went further to evaluate different doses of this agonist. We incubated HUVECs with different concentration of GW3965 (0.05, 0.5, 2.5 μM) for 24 hrs. At the end of incubation, we included LPC for 3 hrs. We found GW3965 reduced IL‐8 secretion in a dose‐dependent manner (Fig. 2C), which was consistent with the mRNA decrease (Fig. 2D). To exclude the possibility that reduction in IL‐8 is a result of cell toxicity by GW3965, we measured cell survival. We found that GW3965 of up to 3 μM did not trigger appreciable cell death (Fig. 2E). We therefore conclude that the inhibition of IL‐8 by LXRs agonist is not caused by cell toxicity, but mediated by intracellular biological changes.

We went further to examine the dependence of GW3965 on LXRs for IL‐8 expression. We first reduced LXR expression by siRNA and then treated the cells with LPC and GW3965. We found the inhibitory effects were largely abolished in LXR knockdown cells (Fig. 2F). These results suggest that GW3965 agonist requires LXRs to suppress LPC‐induced IL‐8 expression in HUVECs.

### LXR agonist reversed LPC‐induced NF‐κB activation and inhibited IL‐8 translation

**Figure 3:**
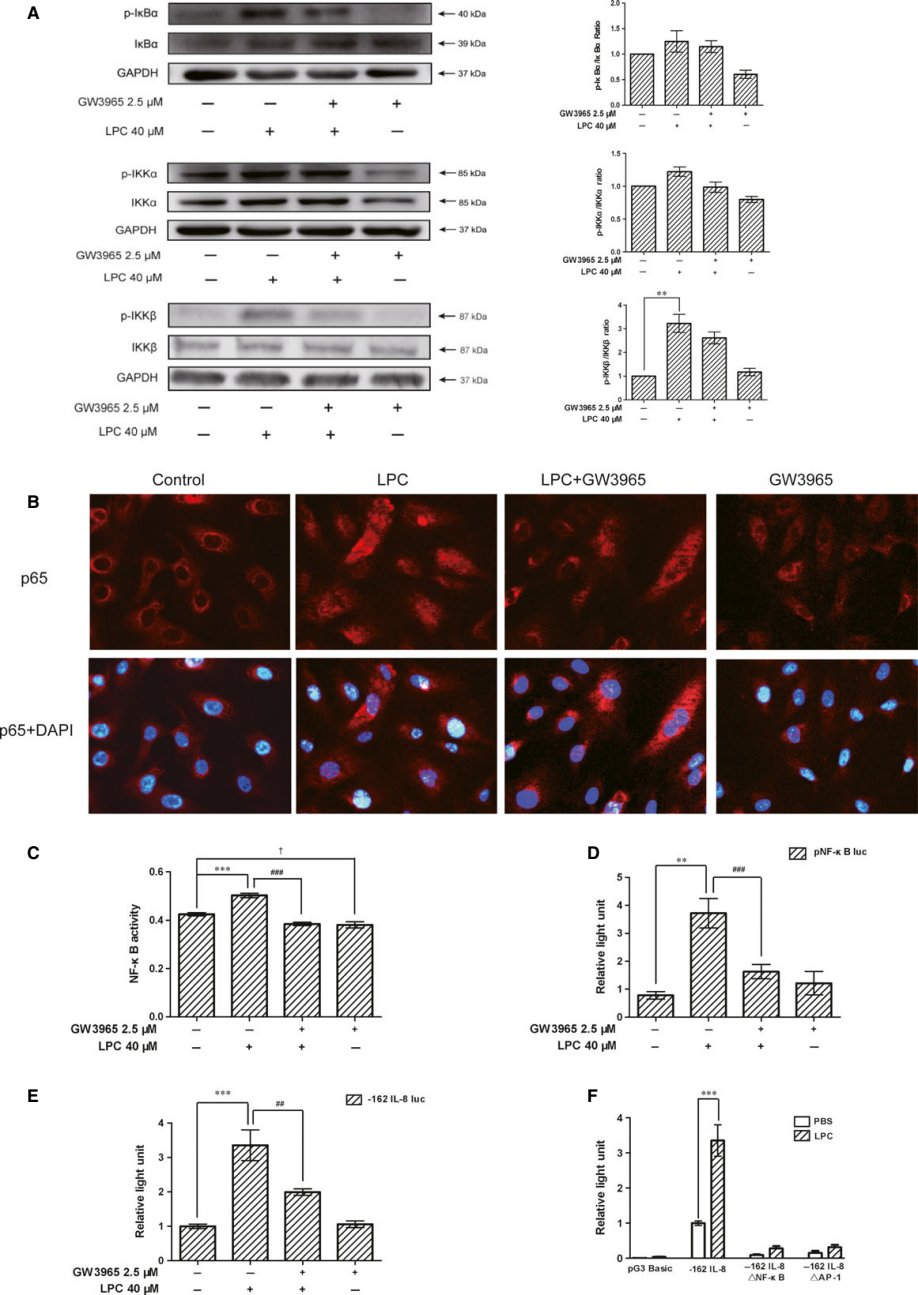
LXR agonist reversed LPC‐induced NF‐κB activation and inhibited IL‐8 translation. (**A**) HUVECs were treated with vehicle or 2.5 μM of GW3965 for 24 hrs and then stimulated with PBS or LPC (40 μM) for 5 min. Relative expression of p‐IKKα was normalized to IKKα, p‐IKKβ was normalized to IKKβ and the expression of p‐IκBα was normalized to IκBα in HUVECs. (**B**) Cells were processed for Immunofluorescence staining to assess the translocation of NF‐κB p65 into the nucleus. (**C**) Nuclear proteins were extracted for the NF‐κB transcription factor activity assay. (**D** and **E**) HUVECs were cotransfected with NF‐κB luciferase reporter plasmid pNF‐κB‐TA‐luc or ‐162 IL‐8 reporter construct, together with the Renilla luciferase reporter as an internal control. Transfected cells were treated with vehicle or 2.5 μM of GW3965 for 24 hrs and then stimulated with PBS or 40 μM of LPC for 3 hrs. The Firefly luciferase activities were analysed by the dual‐luciferase assay and normalized to Renilla luciferase expression. (**F**) HUVECs were transfected with either the empty pGL3‐Basic plasmid, the ‐162 IL‐8 reporter construct or IL‐8 promoter construct containing mutated AP‐1 or NF‐κB sites (ΔAP‐1 or ΔNF‐κB). The cells were treated with or without 40 μM of LPC for 3 hrs. **P < 0.01; ***P < 0.001, compared with the control. ^##^ *P* < 0.01; ^###^ *P* < 0.001, compared with the LPC‐treated group. ^†^ *P* < 0.05 compared with the control. One‐way anova was used to compare the differences.

As the NF‐κB pathway plays a central role in inflammation and chemokine expression, experiments were performed to address whether NF‐κB was involved in the action of the LXR agonist and LPC treatment. Activation of NF‐κB requires the phosphorylation‐ and ubiquitination‐dependent degradation of the IκB proteins; this process is triggered by IKK. We first examined the phosphorylation of IKKα, IKKβ and IκBα. As shown in Figure 3A, LPC treatment led to a significant increase in the phosphorylation of IKKβ. GW3965 treatment did not inhibit phosphorylation of IKKα, IKKβ and IκBα induced by LPC. Immunofluorescence staining showed that p65 subunit of NF‐κB translocated into the nucleus after LPC treatment, and pretreatment with GW3965 antagonized p65 translocation (Fig. 3B). To confirm that the LXR agonist suppressed the activation of the NF‐κB pathway induced by LPC, further studies were performed to evaluate NF‐κB DNA‐binding activity and promoter activity. Figure 3C showed that incubation of HUVECs with LPC‐containing medium increased the NF‐κB DNA‐binding activity, but the effect was suppressed by the LXR agonist treatment.

To investigate the effect of GW3965 on transcriptional inhibition, HUVECs were transfected with the NF‐κB luciferase reporter plasmid pNF‐κB‐TA‐luc or ‐162 IL‐8 reporter plasmid. Figure 3D and E showed the up‐regulation of the NF‐κB activity and IL‐8 promoter induced by LPC was suppressed by the LXR agonist. And more importantly, mutation of NF‐κB and AP‐1 sites in the IL‐8 promoter led to a strong diminishment of LPC‐mediated induction in luciferase activity (Fig. 3F).

### LXR agonist antagonized LPC‐induced IL‐8 induction by inhibiting SUMOylation

**Figure 4:**
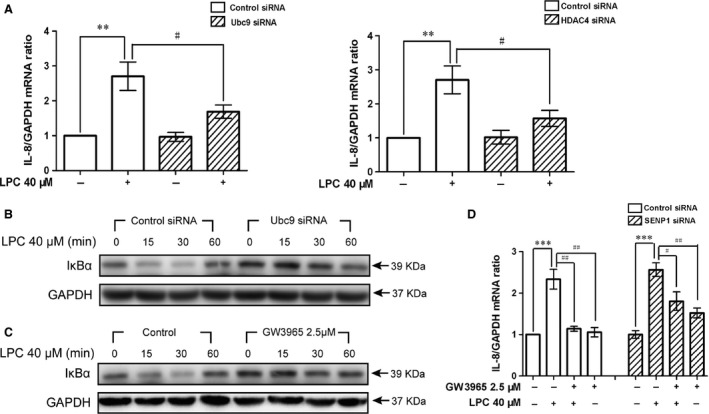
LXR agonist antagonized LPC‐induced IL‐8 expression by inhibiting SUMOylation. (**A**) HUVECs were transfected with siUbc9, siHDAC4 or siControl under the stimulation of LPC (40 μM) for 1 hr. IL‐8 expressed was assessed by real‐time PCR. (**B**) Cells were stimulated by LPC for 0, 15, 30 and 60 hrs after transfecting with siUbc9 or siControl, and IκB levels were detected by immunoblotting. (**C**) HUVECs were stimulated by LPC for 0, 15, 30 and 60 hrs after treating with Vehicle or LXR agonist for 24 hrs and IκB levels were detected by Western Blotting. (**D**) SENP1 knockdown cells were under the stimulation of LPC or vehicle for 1 hr after incubation with or without GW3965. **P < 0.01; ***P < 0.001, compared with the control. ^#^ *P* < 0.01; ^##^ *P* < 0.01 compared with the LPC‐treated group. One‐way anova was used to compare the differences.

To better characterize the relationship between the SUMOylation and the LPC‐induced IL‐8 expression, small ubiquitin‐like modifier (SUMO) E2 ligase Ubc9 and the SUMO E3 ligase HDAC4 were knocked down by specific siRNAs. Silencing of Ubc9 and HDAC4 markedly inhibited LPC‐induced IL‐8 expression (Fig. 4A). These data indicated that SUMOylation was indispensable for the expression of chemokines stimulated by LPC. Inhibitory κBα is known as a SUMO substrate, and the integration of SUMO2/3 appears to enhance the formation of ubiquitin chains on IκBα that promotes its degradation 24. To examine if this known SUMO substrate was affected, Ubc9 silencing experiments were performed. Compared with the control siRNA group, LPC‐induced degradation of IκBα was delayed at early stage of stimulation when Ubc9 was knockdown (Fig. 4B). An obvious defeat was also observed in LPC‐mediated degradation of IκBα under the treatment of LXR agonist (Fig. 4C). GW3965 may stabilize IκBα by inhibiting SUMOylation. Next, we asked the question whether enhancing SUMOylation could rescue the inhibition of GW3965 on LPC‐mediated IL‐8 expression. SUMOylation is reversed by sentrin/SUMO‐specific proteases (SENP) 25. We first silenced SENP1 by siRNAs in HUVECs. We then treated the cells with GW3965 (2.5 μM) for 24 hrs. At the end, we changed the media with LPC (40 μM) for 1 hr. We found that the inhibition of LXRs agonist on IL‐8 mRNA levels was significantly abolished after SENP1 knockdown (Fig. 4D).

### LXRs agonist reversed LPC‐induced monocyte adhesion *via* an IL‐8 dependent mechanism in HUVECs

**Figure 5:**
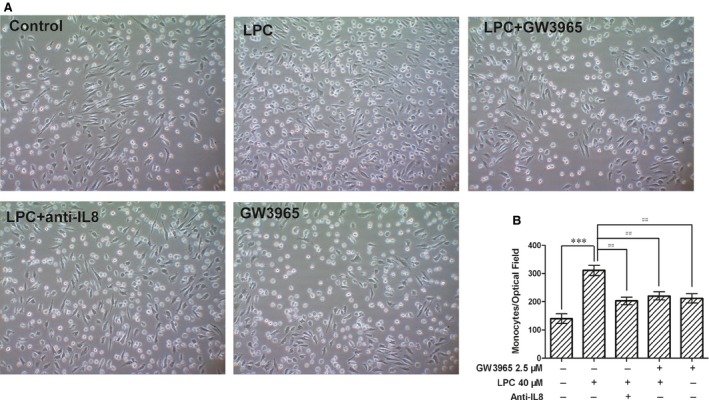
LXR agonist reversed LPC‐induced monocyte adhesion *via* an IL‐8 dependent mechanism in HUVECs. (**A**) An *in vitro* adhesion assay was performed to measure THP‐1 adhesion on vehicle‐stimulated or LPC‐stimulated HUVECs with or without pretreatment with GW3965 for 24 hrs. An IL‐8 neutralizing antibody was used to confirm the role of IL‐8 in THP‐1 adhesion induced by LPC. (**B**) Data shown were pooled from five independent experiments. ***P < 0.001, compared with the control. ^##^ *P* < 0.01, compared with the LPC‐treated group. One‐way anova was used to compare the differences.

To determine the biological function of the LXR agonist on LPC‐induced IL‐8 expression, adhesion of THP‐1 cells to HUVECs was examined. As shown in Figure 5A and B, treatment with LPC enhanced the adhesion of THP‐1 cells to HUVECs. When the HUVECs were pretreated with the LXR agonist (2.5 μM) followed by 40 μM of LPC for 3 hrs, the number of THP‐1 cells adhering to the HUVECs was decreased compared with LPC treatment alone. Moreover, IL‐8 neutralization also attenuated the THP‐1 adhesion induced by LPC. Our results indicate that LXRs activation can significantly decrease monocyte adhesion after LPC treatment and this process is mediated by the down‐regulation of IL‐8.

## Discussion

In this study, we provide evidence that the LXR agonist inhibits LPC‐induced IL‐8 overexpression in HUVECs, which has a biological effect on the firm adhesion of monocytes to the endothelial cells. Lysophosphatidylcholine is a major phospholipid component of ox‐LDL that induces IL‐8 expression and drives monocyte adhesion to endothelial cells *via* Ca^2+^ signaling and NF‐κB pathway, which appear to be responsible for early stage atherogenesis 26, 27. The LPC model in this study provided an opportunity to examine time‐ and dose‐dependent regulatory mechanisms, such as phosphorylation, ubiquitination and SUMOylation of NF‐κB. Previous studies consolidate the role of LXR in atheroprotection 28. In addition to the inhibition of inflammation and promotion of cholesterol efflux, Hayashi *et al*. showed that the activation of LXR inhibits endothelial cell senescence. Therefore, it improves endothelial dysfunction and suppresses the progression of atherosclerosis 19. Treatment of LXRα^−/−^apoE^−/−^ mice with a synthetic LXR ligand ameliorates the cholesterol overload phenotype and reduced atherosclerosis, suggesting that LXRβ plays an important role in this procedure 29. During atherosclerosis regression, LXR is required for maximal effects on plaque CD68^+^ cell expression of CCR7 and monocyte‐derived cell egress 30.

We demonstrated that the LXR agonist reversed LPC‐induced NF‐κB activity by attenuating the degradation of IκBα. Whether IκBα degradation is involved in LXR‐regulated inflammation is subjected to debating. Joseph *et al*. confirmed that the LXR agonist was an effective inhibitor of IκBα expression (3 and 6 hrs after LPS stimulation) by Northern blot analysis. When macrophages are transfected by a dominant‐negative IKK, the inhibitory effect of LXR on the COX‐2 promoter was lost and NF‐κB activity was blocked 9. However, Castrillo *et al*. found LXR activation does not inhibit IκBα degradation (30 min. after LPS stimulation), but inhibits matrix metalloproteinase‐9 promoter activity in macrophages 22. This paradoxical effect of LXR on the inflammatory response is probably because of the selection of time‐points to examine IκBα degradation. In our study, we found that LXR activation prevented NF‐κB binding to DNA and inhibited its translational activity by using an ELISA‐based NF‐κB transcription factor activity assay and a dual‐luciferase reporter assay.

Our experiments suggested that the LXR agonist antagonized LPC‐induced IL‐8 expression *via* inhibiting the SUMOylation. Recent studies have illustrated that proteins containing SUMO‐interaction motif have the capacity to be recognized and SUMO‐modified. Many SUMO‐modified substrates have been found, such as IκBα 31, PPARγ, LXR and even Ubc9 32. As a SUMOylation substrate and a key protein in NF‐κB pathway, IκBα is modified by SUMO2/3 after stimulation of tumour necrosis factor‐α. And SUMOylated IκBα promotes the degradation of IκBα and optimal control of NF‐κB pathway 24. In our study, we found the activation of LXR suppressed the degradation of IκBα and inhibited NF‐κB activity. Our experiment shows that the activation of LXR may delay IκBα degradation by inhibiting SUMOylation. These results suggest a new crosstalk between LXR and NF‐κB.

However exploratory, this study has some limitations. First of all, our experiments were performed *in vitro*, and thus it is not clear that these would have a translational capacity. Besides, the substrate of SUMOylation and the reason why LXR can affect post‐translational modifications were not demonstrated clearly, further studies are therefore required to delineate the underlying mechanisms.

## Conflict of interest

The authors confirm that there are no conflicts of interest.
